# Physical activity levels and weight control status by body mass index, among adults – National Health and Nutrition Examination Survey 1999–2004

**DOI:** 10.1186/1479-5868-5-25

**Published:** 2008-05-01

**Authors:** Judy Kruger, Michelle M Yore, Harold W Kohl

**Affiliations:** 1 Physical Activity and Health Branch, The Division of Nutrition, Physical Activity, and Obesity, Centers for Disease Control and Prevention, Atlanta, Georgia, USA; 2 University of Texas School of Public Health, Michael & Susan Dell Center for Advancement of Healthy Living, Division of Epidemiology, Austin, Texas, USA

## Abstract

**Background:**

Adequate levels of physical activity can assist with weight control efforts, however, only a modest number of national studies have examined the physical activity patterns by weight control status. This article aims to describe patterns of physical activity among men and women who reported engaging in weight control practices.

**Methods:**

Data from the National Health and Nutrition Examination Survey (1999–2004) were used. The sample included 14,388 adults (aged ≥ 18 years), with measured weights and heights from which body mass index (BMI) (weight/height^2^; kg/m^2^) was calculated. Analyses were performed to describe the prevalence of engaging in levels of physical activity (< 150–630 minutes/week) by three levels of weight control status (trying to lose weight, trying to maintain weight, and not trying to lose/maintain weight). We also examined the association between physical activity level and weight control status by BMI.

**Results:**

The prevalence of low levels of physical activity (< 150 minutes/week) was highest among those not trying to lose/maintain weight (77.7% men, 81.2% women), than those trying to lose, or maintain weight (64.2%–59.7% men, 68.1%–66.7% women). Significantly more men than women engaged in higher volumes of physical activity (p < 0.001). Among overweight men, those trying to lose weight were more likely to engage in 150–420 minutes/week (OR = 2.2, 95%CI 1.8–2.9) than those not trying to lose/maintain weight. Similarly, overweight women who were trying to lose weight were more likely to engage in 150–420 minutes/week (OR = 2.8, 95%CI 2.1–3.7) than were to those not trying to lose/maintain weight.

**Conclusion:**

Despite people's intentions to lose or maintain their weight, the majority of adults do not engage even in the minimum recommended level of physical activity. However, the prevalence of engaging in high levels of physical activity (150–420 minutes/week) was highest among those trying to lose or maintain weight than were with those not trying to lose/maintain weight. Regardless of weight control status, all adults should be encouraged to engage in regular physical activity.

## Background

The literature suggests that having a body mass index (BMI) of ≥ 30 kg/m^2 ^is a factor that contributes to many negative health conditions, premature death, and disability [1]. Although physical activity plays a major role in maintenance of energy balance, few adults with BMI of ≥ 30 kg/m^2 ^engage in regular physical activity [2]. Although cause and effect has not been documented, one possible reason for this is that it is more difficult for heavier people to move at the same exercise intensity as those of normal weight [3,4]. It has also been suggested that excess body weight poses additional challenges in terms of temperature regulation and the dissipation of body heat while exercising, as well as problems related to the buildup of sweat which may cause chafing due to movement [5]. When people chose to become physically active, the literature suggest that people begin at a low intensity level [6,7], which may contribute to an active lifestyle and be easier for obese people to perform, but expend fewer calories than higher intensity activity for people of the same body size. Moreover, when previously sedentary people are prescribed an intensity that they perceive to be difficult, they are less likely to continue participation [8].

Increasing the adoption and maintenance of regular physical among adults is a major challenge. In addition to perceived stress, King and colleagues [9] found that BMI > 27 kg/m^2 ^and low fitness levels were predictive of poor adherence to long-term physical activity. Although it is not clear which factors are most critical to the adoption or maintenance of activity, research on predictors of successful weight control has shown that the overall attrition rate for a 16-week program is roughly 30% for those who engaged in physical activity sufficient to expend 300–500 kcal/day [10] or > 2000 kcal/week [11]. To date, there is limited research supporting the maintenance of high volumes of physical activity in population-based studies.

Currently, the specific physical activity recommendations in the 2005 *Dietary Guidelines for Americans *are to reduce the risk of chronic disease, to manage body weight and prevent weight gain, and to sustain weight loss [12]. The recommended amount of physical activity varies between 30–90 minutes on most, preferably all, days of the week. Findings from the literature suggest that in 2001, only 31.6% of U.S. adults met the minimal recommended amount of 30 minutes of moderate-intensity physical activity most days of the week [13]. Moreover, one study found that participation in high levels of physical activity (e.g., 60 minutes of at least moderate-intensity physical activity throughout each day) even among those who report using exercise to lose weight is only 19.2% [14]. Few national data describe patterns of physical activity by BMI. The objectives of this study, which used data from the 1999–2004 NHANES, were to examine the prevalence of physical activity by weight control category (weight loss, weight maintenance, or not trying to lose/maintain weight) among men and women, and associations between physical activity and weight control status across different levels of BMI.

## Methods

The National Health and Nutrition Examination Survey (NHANES) is undertaken by the National Center for Health Statistics, Centers for Disease Control and Prevention and assesses health and nutritional status of adults and children in the United States [15]. NHANES is unique in that it combines interviews and physical examinations. Data are released on public use data files every two years. In the current study, data from three cycles (1999–2000; 2001–2002 and 2003–2004) were combined in order to increase sample size and analytic options. Because data items collected in all combined years were comparable in wording and methods, they could be concatenated to form a single file (interview sample size of 31,126).

From the initial sample size of 31,126 we excluded those who were < 18 years of age, pregnant women, and those with unknown relevant information (e.g., physical activity, demographic characteristics, and weight control status). The final analytic sample consisted of 14,388 respondents. In 1999–2000, the overall exam response rate for those interviewed was 76.3%; in 2001–2002 it was 79.6%, and in 2003–2004 it was 75.6%.

Physical activity was assessed by asking respondents about their participation in specific physical activities. Respondents were asked, "Over the past 30 days, did you do any vigorous activities for at least 10 minutes that caused heavy sweating, or large increases in breathing or heart rate? Some examples are running, lap swimming, aerobics classes or fast bicycling." Those who reported "yes" were asked, " [Over the past 30 days] what vigorous activities did you do?" Respondents were handed a card with a list of 24 vigorous activities and an "other" category. They were then asked about the frequency (e.g., how many times per day, per week, or per month) and duration (e.g., how long in terms of minutes or hours) they performed these activities. In a similar manner, respondents were also asked about their participation in moderate-intensity physical activity: " [Over the past 30 days], did you do moderate activities for at least 10 minutes that cause only light sweating or a slight to moderate increase in breathing or heart rate? Some examples are brisk walking, bicycling for pleasure, golf, or dancing." Individuals who reported that they had engaged in moderate-intensity activity were asked to report the frequency and duration of any of the 32 moderate activities.

The total minutes spent on all moderate or vigorous activities in the past 30 days was used to calculate average minutes (volume) of weekly leisure-time physical activity of moderate intensity or greater. Physical activity was defined on four mutually exclusive levels: (1) < 150 minutes/week, (2) 150–420 minutes/week (i.e., equivalent to 30 minutes on 5 days of the week), (3) 420–630 minutes/week (i.e., equivalent to 60 minutes on 7 days of the week); or (4) ≥ 630 minutes/week (i.e., equivalent to 90 minutes on 7 days of the week).

Weight control status was assessed with the following questions. Respondents were asked, "During the past 12 months, have you tried to lose weight?" This question was skipped in those who reported an intentional weight loss from their current weight of ≥ 10 lb to their weight one year ago. Respondents were categorized as trying to lose weight if they lost ≥ 10 lb in the past year. Additionally, all respondents were asked, "During the past 12 months, have you done anything to keep from gaining weight?" After excluding those who reported trying to lose weight, respondents were categorized as trying to maintain weight if they answered they were trying to keep from gaining weight during the past 12 months. We therefore created three mutually exclusive categories of weight control maintenance status: (1) those reporting they were trying to lose weight, (2) those trying to maintain weight, and (3) those not trying to lose or maintain weight.

Measured height and weight were collected in the NHANES Mobile Examination Center; standing height using a fixed stadiometer and body weight was measured on a Toledo digital scale. We calculated BMI as weight (kilograms) divided by squared height (meters) and grouped respondents into three categories on the basis of the National Heart, Lung, Blood Institute guidelines [16]: normal weight (18.5 to < 25.0 kg/m^2^), overweight (25.0 to 29.9 kg/m^2^), or obese (≥ 30.0 kg/m^2^).

Stratified analyses were conducted to compare the prevalence of engaging in the three weight control categories among men and women by age group, race/ethnicity, education, and BMI. To account for the complex sampling design, SUDAAN version 9.0 (Research Triangle Institute, Research Triangle Park, NC) was used. Age-adjusted prevalence estimates were calculated using the year 2000 U.S. standard population [17] to describe physical activity levels among each weight control category (lose weight, maintain weight, or not trying to lose/maintain weight). Chi-square was used to test statistical significance between weight control categories, demographics and physical activity level. Separate logistic regression models were calculated for men and women with physical activity (yes or no for the various levels of activity) as the dependent variable. We calculated the odds ratios (OR) and 95% confidence intervals (95% CI) for the association between physical activity level and weight control status across different levels of BMI (normal weight, overweight and obese). The referent group was those not trying to lose or maintain weight. Multivariate models were adjusted for the following covariates: age, race/ethnicity, and education.

## Results

Slightly more than one-third of men and women trying to lose weight were between 45 and 64 years of age (Table 1). Among those trying to lose weight, 73.9% of men and 70.6% of women were non-Hispanic whites. More than 55% of men and women who were trying to lose weight had more than a high school education. More than 81% of those trying to maintain weight were non-Hispanic white. Among those trying to maintain weight, 69.2% of men and 66.3% of women had more than a high school education. More than 44% of those not trying to lose/maintain weight had more than a high school education. Roughly 41% of those trying to lose weight were obese and more than 24% of those trying to maintain weight were obese.

**Table 1:** Characteristics of U.S. adults (≥ 18 years) by weight control status

	**Weight control status**^a^											
	**Lose weight**				**Maintain weight**				**Not trying to lose/maintain weight**			
	Men		Women		Men		Women		Men		Women	

	N^b^	%^c^	N^b^	%^c^	N^b^	%^c^	N^b^	%^c^	N^b^	%^c^	N^b^	%^c^

**Total**	2,618	38.6	3,748	55.3	618	10.0	553	8.9	3,936	51.4	2,915	35.7
**Age group (years)**^d^												
18–29	562	19.5	953	20.8	140	16.6	124	16.5	1,149	25.9	695	18.2
30–44	607	31.0	932	30.5	138	30.4	142	31.6	911	32.9	609	28.4
45–64	814	35.8	1,170	35.5	180	37.3	139	31.0	929	27.1	703	29.2
**≥**65	635	13.7	693	13.3	160	15.7	148	20.9	947	14.2	908	24.2
**Race/ethnicity**^d^												
Mexican-American	614	7.7	899	6.9	102	4.5	110	5.3	1,027	9.3	723	7.1
Non-Hispanic white	1,328	73.9	1,736	70.6	372	80.5	346	83.0	1,755	68.4	1,300	68.0
Non-Hispanic black	481	8.9	811	11.9	104	7.3	67	6.2	843	11.2	637	13.0
Other	195	9.5	302	10.7	40	7.6	30	5.6	311	11.1	255	12.0
**Education**^d^												
< High school	769	16.7	1,132	18.5	113	9.5	111	12.1	1,643	26.9	1,168	27.3
High school	610	24.9	946	26.9	138	21.3	117	21.6	992	29.2	725	27.0
> High school	1,237	58.5	1,666	54.6	367	69.2	323	66.3	1,294	43.9	1,018	45.6
**Body Mass Index**^d^												
Normal	421	14.4	909	27.4	155	24.9	251	51.3	1,818	45.1	1,298	49.0
Overweight	1,114	43.9	1,170	31.4	306	49.5	152	25.2	1,446	36.6	871	26.6
Obese	1,083	41.8	1,669	41.3	157	25.6	150	23.6	672	18.3	746	24.4

^a ^Weight control status refers to: Lose weight refers to those who self-reported trying to lose weight or who lost ≥ 10 pounds in the past year; maintain weight refers to those who self-reported trying not to lose weight and were trying to maintain weight.

^b ^Unweighted sample size.

^c ^Percent is weighted. Prevalence estimates are age-adjusted (except for those by age group) to the 2000 U.S. standard population using the age group found in the table.

^d ^Statistically significant (p < 0.001) by gender.

**Figure 1:**
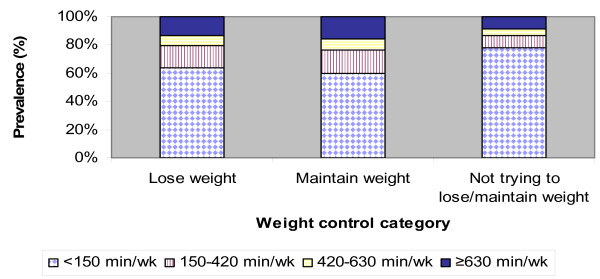
Prevalence of physical activity (min/wk) by weight control category among men.

Among men, the prevalence of low levels of physical activity (< 150 minutes/week) was highest among those not trying to lose/maintain weight, compared to those trying to lose, or maintain weight (Figure 1). Among men in all weight control groups, there was a lower prevalence of reporting physical activity as the total number of minutes increased from 150–630 minutes/week. Among those who were trying to lose weight, 6.7% reported engaging in high levels of physical activity (420–630 minutes/week) which was statistically different from those trying to maintain weight (8.3%), or those not trying to lose/maintain weight (4.9%) (p < 0.001).

**Figure 2:**
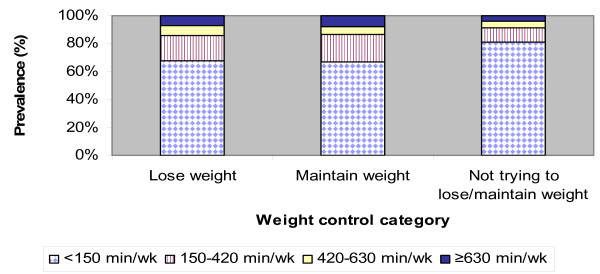
Prevalence of physical activity (min/wk) by weight control category among women.

Among women not trying to lose/maintain weight, the prevalence of engaging in low levels of physical activity (< 150 minutes/week) was significantly different from those trying to lose, or maintain weight (p < 0.001) (Figure 2). Among those who were trying to lose weight, a greater proportion of women engaged in the minimal recommended amount of physical activity (150–420 minutes/week), compared to those trying to maintain weight, or not trying to lose/maintain weight (p < 0.001). Significantly more men than women engaged in higher volumes of physical activity (p < 0.001).

Among men who were overweight, those who were trying to lose weight, were significantly more likely to engage in 150–420 minutes/week than those not trying to lose or maintain weight (adjusted OR = 2.2, 95%CI 1.8–2.9) (Table 2). A similar pattern among women who were overweight was noted. Those who were trying to lose weight were significantly more likely to engage in 150–420 minutes/week than those not trying to lose or maintain weight (adjusted OR = 2.8, 95%CI 2.1–3.7). Obese men and women who were trying to lose weight were significantly more likely to be regularly active at recommended levels of physical activity, compared with those not trying to lose/maintain weight.

**Table 2:** The association between physical activity level and weight control status by BMI.

	**Physical activity level (min/wk)**							
	**Men**				**Women**			
	**< 150**	**150–420**	**420–630**	**≥ 630**	**< 150**	**150–420**	**420–630**	**≥ 630**
**BMI**	**OR**^a^**(95% CI)**	**OR**^a^**(95% CI)**	**OR**^a^**(95% CI)**	**OR**^a^**(95% CI)**	**OR**^a^**(95% CI)**	**OR**^a^**(95% CI)**	**OR**^a^**(95% CI)**	**OR**^a^**(95% CI)**
**Normal weight**								
Trying to lose	0.6 (0.4–0.8)	1.8 (1.3–2.3)	1.6 (1.1–2.3)	1.6 (1.1–2.3)	0.5 (0.4–0.7)	1.9 (1.5–2.4)	1.7 (1.2–2.4)	2.0 (1.4–3.0)
Trying to maintain	0.3 (0.2–0.5)	3.2 (2.2–4.6)	2.3 (1.6–3.2)	1.9 (1.2–3.1)	0.5 (0.4–0.8)	1.9 (1.2–2.9)	1.5 (1.0–2.4)	2.1 (1.4–3.3)
Not trying to lose/maintain	1.0	1.0	1.0	1.0	1.0	1.0	1.0	1.0
**Overweight**								
Trying to lose	0.5 (0.4–0.6)	2.2 (1.8–2.9)	1.9 (1.5–2.4)	1.9 (1.5–2.5)	0.4 (0.3–0.5)	2.8 (2.1–3.7)	1.6 (1.1–2.3)	1.8 (1.1–2.9)
Trying to maintain	0.5 (0.4–0.8)	1.9 (1.3–2.8)	1.6 (1.1–2.4)	1.7 (1.2–2.5)	0.4 (0.3–0.7)	2.6 (1.5–4.4)	1.4 (0.6–3.1)	1.8 (0.5–6.3)^b^
Not trying to lose/maintain	1.0	1.0	1.0	1.0	1.0	1.0	1.0	1.0
**Obese**								
Trying to lose	0.4 (0.3–0.6)	2.4 (1.7–3.3)	2.3 (1.5–3.4)	2.6 (1.5–4.6)	0.4 (0.3–0.6)	2.6 (1.7–4.0)	2.5 (1.3–4.9)	5.6 (1.2–5.7)
Trying to maintain	0.4 (0.3–0.7)	2.3 (1.4–3.9)	3.4 (1.9–6.3)^b^	3.9 (2.1–7.4)^b^	1.1 (0.5–2.3)	0.9 (0.4–1.9)	0.7 (0.2–2.5)	1.5 (0.3–6.5)^b^
Not trying to lose/maintain	1.0	1.0	1.0	1.0	1.0	1.0	1.0	1.0

^a ^Separate logistic regression equations for each physical activity variable. Dependent variables: < 150 min/week (yes/no), 150–420 min/week (yes/no), 420–630 min/week (yes/no), ≥ 630 min/week (yes/no). Models are adjusted for age, race/ethnicity, and education.

^b ^Estimates may be unstable because sample size is < 50.

## Discussion

Differences in physical activity level among those trying to lose, maintain or not lose/maintain weight were evident. Findings from these national data suggest that the prevalence of engaging in any physical activity is higher among those actively engaging in weight control practices (trying to lose or maintain weight) compared with those not trying to lose/maintain weight. Moreover, men had a significantly higher prevalence than women in engaging in higher volumes of physical activity. Our gender-specific findings are similar to those by Serdula and colleagues [18] who examined both nutrition and physical activity strategies and showed that 42.3% of men and 36.8% of women reported engaging ≥ 150 minutes/week of leisure-time physical activity.

To help manage body weight and prevent gradual weight gain, the 2005 *Dietary Guidelines *[12] suggest that adults engage in approximately 60 minutes of moderate to vigorous-intensity activity on most days of the week while not exceeding caloric intake requirement. We found that the prevalence of being active at higher volumes (e.g., 420–630 minutes/week or ≥ 630 minutes/week) was low among both men and women across all weight control groups. Our findings suggest that despite people's intentions to lose or maintain weight, a large proportion of adults are not regularly active even at minimum recommended levels of physical activity. Because participation in physical activity has been shown to result in modest weight loss [19,20], adults are encouraged to increase their overall total time being physically activity [3].

The results presented from NHANES examine the association between physical activity level and reported weight control status across different levels of BMI. When examining total volume of regular physical activity, our results show that obese men and women who are trying to lose weight were significantly more likely to engage in recommended levels of physical activity compared to those not trying to lose/maintain weight. The American College of Sports Medicine suggests that overweight and obese adults engage in 150 minutes/week and to progress to higher amounts of exercise (e.g., > 200 minutes/week) to facilitate long-term maintenance of weight loss [21]. Previous findings from voluntary enrollees of the National Weight Control Registry suggest that long-term weight loss maintenance requires regular physical activity to be successful in weight control [22,23]. Regardless of BMI status, it has been suggested that successful and sustainable weight loss and weight maintenance strategies require a gradual increase in the volume of physical activity [21].

Regular physical activity is a key factor in weight loss maintenance. The 2005 *Dietary Guidelines *suggest that for previously overweight or obese adults to maintain weight loss, they need to engage in about 60 to 90 minutes of moderate-intensity physical activity per day [12]. Prior to encouraging people to engage in higher levels of physical activity, it may be more feasible to encourage sedentary adults to engage in the minimal recommended amount of physical activity. Adults trying to control their weight can be encouraged to increase their total energy expenditure by engaging in prolonged durations over time, increasing the intensity of the activities pursued, or combining these two approaches [19].

## Limitations

This study is limited by several factors. First, data are cross-sectional, and no firm conclusions can be made regarding causal relationships between physical activity, weight control behavior, and BMI. Second, the physical activity and intended weight loss data are self-reported and subject to potential misclassification bias, although measured height and weight were used in this analysis. Responses may be an over- or underestimation of actual behavior because respondents may be prone to provide socially desirable responses (e.g., engaged in higher levels of physical activity, or reported trying to lose weight if they were obese). Third, there may be bias due to the time frame phrased in the questions. The questions on weight control status referred to behavior during the past 12 months and the questions on physical activity referred to behavior during the past 30 days. Therefore, we do not know how long respondents engaged in their current physical activity pattern in the last 12 months. It was also not possible to calculate the frequency in terms of days per week respondents reported being active. Fourth, the weight history and physical activity questions from NHANES have not been validated to date. It was not possible to validate respondents self-reported weight loss of 10 or more pounds. Fifth, the sample sizes of those trying to maintain weight were small, and thus had more variability.

However, use of the NHANES data has several strengths. First, NHANES is a large national dataset, which allows us to have a large sample size and examine many inter-related variables. Second, we combined NHANES data over 6 years to yield an even richer data source. Third, NHANES data allowed us to examine BMI based on measured weight and height data, which are more accurate than those based on self-reported data [24].

## Conclusion

Despite efforts by U.S. adults to control their weight, the results from this study suggest that the prevalence of engaging in the minimum recommended level of physical activity is low. Although, the prevalence of engaging in high levels of physical activity was highest among those trying to lose, or maintain weight compared with those not trying to lose/maintain weight, increased efforts are needed to encourage all adults to reduce the amount of time engaged in low-intensity activities such as sitting. Regardless of body size, adults should be encouraged to adopt an active lifestyle and engage in ≥ 30 minutes of physical activity on most days of the week.

## List of abbreviations

OR: Odds ratios; 95% CI: 95% confidence intervals.

## Competing interests

The authors declare that they have no competing interests.

## Authors' contributions

JK carried out the analysis plan, interpretation, and writing of manuscript. HWK aided in the analysis plan, interpretation, and writing of manuscript; MMY performed the statistical analysis, aided in the interpretation of results, writing and editing of manuscript. All authors read and approved the final manuscript.
